# Omicron related COVID-19 prevention and treatment measures for patients with hematological malignancy and strategies for modifying hematologic treatment regimes

**DOI:** 10.3389/fcimb.2023.1207225

**Published:** 2023-10-19

**Authors:** Wenjing Guo, Yizhou Zheng, Sizhou Feng

**Affiliations:** ^1^ State Key Laboratory of Experimental Hematology, National Clinical Research Center for Blood Diseases, Haihe Laboratory of Cell Ecosystem, Institute of Hematology & Blood Diseases Hospital, Chinese Academy of Medical Sciences & Peking Union Medical College, Tianjin, China; ^2^ Tianjin Institutes of Health Science, Tianjin, China

**Keywords:** SARS-CoV-2, Omicron, hematological malignancy, treatment, immunocompromise

## Abstract

The Omicron variant of SARS-CoV-2 has rapidly become the dominant strain worldwide due to its high transmissibility, although it appears to be less pathogenic than previous strains. However, individuals with hematological malignancy (HM) and COVID-19 remain susceptible to severe infection and mortality, especially those with chronic lymphocytic leukemia (CLL) and those undergoing chimeric antigen receptor T-cell (CAR-T) treatment. Hematologists should thoroughly assess the severity of the patient’s hematological disease and the potential risk of SARS-CoV-2 infection before initiating chemotherapy or immunosuppressive treatment. Vaccination and booster doses are strongly recommended and patients with a poor vaccine response may benefit from long-acting COVID-19 neutralizing monoclonal antibodies (such as Evusheld). Early use of small molecule antiviral drugs is recommended for managing mild COVID-19 in HM patients and those with severe immunodeficiency may benefit from SARS-CoV-2 neutralizing monoclonal antibody therapy and high-titer COVID-19 convalescent plasma (CCP). For moderate to severe cases, low-dose glucocorticoids in combination with early antiviral treatment can be administered, with cytokine receptor antagonists or JAK inhibitors added if the condition persists or worsens. In the treatment of hematological malignancies, delaying chemotherapy is preferable for CLL, acute leukemia (AL), and low-risk myelodysplastic syndrome (MDS), but if the disease progresses, appropriate adjustments in dosage and frequency of treatment are required, with the avoidance of anti-CD20 monoclonal antibody, CAR-T and hematopoietic stem cell transplantation (HSCT). Patients with chronic myelocytic leukemia (CML) and myeloproliferative neoplasms (MPNs) can continue current treatment. What’s more, non-drug protective measures, the development of new vaccines and antiviral drugs, and monitoring of mutations in immunocompromised populations are particularly important.

Severe acute respiratory syndrome coronavirus 2 (SARS-CoV-2) is a single stranded RNA virus with a very high mutation frequency in its genome (Vitiello et al., 2022). While the majority of these mutations appear to have no impact on the virus’s function, a few confer a replication advantage upon the virus (Tuekprakhon et al., 2022), leading to its widespread circulation and making it a variant of concern (VOC). Since December 2019, a variety of VOCs such as alpha, beta, gamma, delta and omicron have emerged in the COVID-19 pandemic. In November 2021, Omicron (B.1.1.529) variant and its sublineages such as Omicron BA.1.1, BA.2, BA.5, were first identified in South Africa. Multiple mutations in the receptor binding domain (RBD) and N-terminal domain of the spike (S) protein contribute to Omicron’s immune evasion capability (Cao et al., 2022), high affinity for the ACE2 receptor (Tuekprakhon et al., 2022) and high replication rate in the bronchus (Hui et al., 2022). These mutations have made the SARS-CoV-2 the dominant strain globally, with BQ.1.1 (BA. 5 subtype) and XBB (BA. 2 subtype) being of particular concern due to their rapidly increasing prevalence (Imai et al., 2023). However, further research has found that Omicron exhibits a lower replication rate (Hui et al., 2022) and pathogenicity (Yuan et al., 2022) in the lung parenchyma than other VOCs. In light of universal vaccination and the emergence of new therapeutic drugs, outcomes in COVID-19 patients during the Omicron era have significantly improved compared to before, with most individuals experiencing a mild and self-limiting course of the disease (Innes et al., 2020). Nevertheless, patients with hematological malignancies (HM) remain at a higher risk of infection and death than other populations due to impaired autoimmune function and treatment-related immunosuppression. These patients often face the dilemma of whether to reduce or stop medication or delay treatment, as well as the particularity of COVID-19 associated treatment. This review presents the latest advances in epidemiology, prevention and control strategies, and treatment measures for patients with HM and COVID-19 in the Omicron era, providing valuable insights into the management of this complex population.

## 1 Epidemiological characteristics

Based on various research findings, the mortality rate of HM patients who presented with COVID-19 before the Omicron epidemic ranged from 13.8% to 40.7% (Cattaneo et al., 2020; Dai et al., 2020; Passamonti et al., 2020; Wood et al., 2020; Pagano et al., 2021; Yigenoglu et al., 2021), significantly higher than that of the general population (0.1%~9.4%) (Pagano et al., 2021) and of solid tumor patients (6%~14.93%) (Wang et al., 2021). Due to the rising COVID-19 vaccination rate, the development of therapeutic drugs and the decrease in the virulence of Omicron, the mortality rate of COVID-19 in the general population has significantly dropped to 0.11%~0.56% (Nyberg et al., 2022; Smith et al., 2022), and that of HM patients with COVID-19 has dropped to approximately 2.2%~9.1% (Ali EA. et al., 2022; Ali EaH. et al., 2022; Blennow et al., 2022; Bronstein et al., 2022; Della Pia et al., 2022; Kohn et al., 2022; Lee M. et al., 2022; Martin-Onraët et al., 2022; Pagano et al., 2022; Pinato et al., 2022; Rasmussen et al., 2022; Shaw et al., 2022; Taenaka et al., 2022; Zerbit et al., 2022; Zhu et al., 2023).

The risk of death from SARS-CoV-2 infection varies across different types of HM and has slightly changed during the Omicron era compared to before. Many studies have confirmed that patients with myeloid malignancies such as acute myeloid leukemia (AML) and myelodysplastic syndrome (MDS) have the highest mortality rate after infection (40%~46.4%) (Pagano et al., 2021; Marchesi et al., 2022) between 2019 and 2021, before the Omicron epidemic. Even patients with acute promyelocytic leukemia (APL) have a high incidence of severe pneumonia (Palanques-Pastor et al., 2021; Marchesi et al., 2022). However, Pagano et al. (2022) have indicated that after vaccination, there is no significant difference in mortality rates between AML and MDS patients developing breakthrough COVID-19 infections (9.2%) and other types of HM patients. This finding may be associated with the fact that most patients are diagnosed with Omicron-related COVID-19 and the seroconversion rate of AML patients after vaccination is relatively higher.

Patients with acute lymphoblastic leukemia (ALL) are also at a higher risk of death after SARS-CoV-2 infection, mainly due to their advanced age, rapid disease progression and drug-related adverse reactions (Pagano et al., 2021), but there is limited research available on the mortality risk of ALL patients in the Omicron era. Due to B-cell or T-cell dysfunction, hypogammaglobulinemia and advanced age, the risk of infection and death for chronic lymphocytic leukemia (CLL) patients is still high during the Omicron period compared to the previous period (13.9%~23.0% vs 20%~33%) (Bronstein et al., 2022; Niemann et al., 2022). The mortality of lymphoma patients infected with Omicron was similar to the overall mortality of contemporary HM patients (5.3%~8.8%) (Bronstein et al., 2022; Della Pia et al., 2022; Kohn et al., 2022), which was significantly lower than before. Studies have revealed that patients with chronic myeloid leukemia (CML) and myeloproliferative neoplasm (MPN) tend to experience milder symptoms and exhibit a lower risk of death following SARS-CoV-2 infection (Ali EA. et al., 2022; Ali EaH. et al., 2022). Notably, the tyrosine kinase inhibitors (TKI) and Janus tyrosine kinase (JAK) inhibitors, common therapeutic agents employed in the management of CML and MPN, possesses antiviral, immunomodulatory and endothelial protective properties, contributing positively to the prognosis of these patients (García-Suárez et al., 2020; Delgado and Torres, 2022) (see part III for details).

In conclusion, despite the notable improvement in the prognosis of COVID-19 patients (including those with HM) during the Omicron period, HM patients remain a high-risk population. The outcomes of SARS-CoV-2 infection vary widely among patients with distinct types of HM. Prior to the emergence of Omicron, AML patients exhibited the highest mortality risk, while during the Omicron period, the highest risk was observed in CLL patients, which may be associated with factors such as the vaccine response rate, the treatment drugs for blood diseases, and the lack of research focused on hematologic patients with other specific malignancies.

## 2 How to prevent SARS-CoV-2 infection?

### 2.1 Vaccination

During the epidemic of Omicron, vaccination is still the most effective strategy to reduce the infection rate and mortality (Leuva et al., 2022). HM patients should be vaccinated if their condition permits. In a retrospective multicenter study in Europe (Pinato et al., 2022), which analyzed a cohort of 3473 cancer patients (14.3% HM) diagnosed with COVID-19 during the pre-vaccination, alpha-delta and omicron phases respectively, the vaccination rate during the Omicron period was 87.6% (297/339), whereas that during the alpha-delta period was only 28.0% (256/915). Notably, the hospitalization rate and 28-day mortality of HM patients infected with SARS-CoV-2 in the omicron period were 52.5% and 4.7% respectively, which were significantly lower than those in pre-vaccination (62.4%, 30.4%) and alpha-delta stage (44.1%, 19.7%) (*P*<0.0001). But for cancer patients who were not vaccinated, there was no significant difference in COVID-19 related hospitalization rate (42.9% vs 40.8%) and mortality (27.5% vs 28.0%) during the Omicron and alpha-delta-phases.

However, due to impaired humoral immunity and reduced ability to maintain memory immune response in HM patients, the seroconversion rate (the production of anti-SARS-CoV-2 spike (S) protein antibody) after receiving two doses of vaccine is significantly lower than that of solid tumor patients (59% vs 85%) (Fendler et al., 2021). Moreover, the vaccine efficacy in 3-6 months after vaccination is also lower than that of solid tumor patients and healthy individuals (27.4% vs 49.8% vs 61.4%) (Lee LYW. et al., 2022). Therefore, the risk of breakthrough infections in HM patients after vaccination is higher than that in other populations (Song et al., 2022). In response to this, the Food and Drug Administration (FDA) approved the use of a booster dose (the third dose) in August 2021 for people with immune impairment (Greenberger et al., 2021). Patients with low neutralizing antibody titers or no response after one booster shot can benefit from another vaccination after three months (the fourth dose) (Krekeler et al., 2022; Mai et al., 2022). Researches have shown that the delivery of booster doses can significantly improve the seroconversion rate (78.8%), antibody titers (comparable to healthy adults) and neutralizing ability against Omicron (Haggenburg et al., 2022), as well as improve the prognosis of HM patients (Blennow et al., 2022). A multicenter prospective study (Minoia et al., 2023) involving 82 HM patients contracted SARS-CoV-2 during the Omicron infection period found that receiving 2 doses (aOR: 0.06, 95%CI: 0.01-0.22, *P*=0.006) or 3-4 doses (aOR: 0.02, 95%CI: 0.01-0.21, *P*=0.001) of the vaccine could reduce the incidence of lung failure. Approximately 44%~56% (Greenberger et al., 2021; Mai et al., 2022; Ollila et al., 2022) of HM patients whose serological response remains negative after full vaccination may experience seroconversion after the booster shot, but it remains hard for patients who have received anti-CD20 monoclonal antibody (Mai et al., 2022) and chimeric antigen receptor T-cell (CAR-T) treatment (Haggenburg et al., 2022) within six months to benefit from the booster shot.

### 2.2 SARS-CoV-2 neutralizing monoclonal antibodies

SARS-CoV-2 neutralizing monoclonal antibodies (NmAbs), exemplified by Evusheld (AZD7442), can bind to the spike protein’s RBD, hindering its interaction with the human ACE2 receptor. Administering NmAbs is an effective passive immunization method, offering protection for seronegative COVID-19 patients (lack anti-SARS-CoV-2 antibody) (RECOVERY Collaborative Group, 2022a). Injection with Evusheld before and after SARS-CoV-2 exposure has demonstrated promising results in preventing infection, avoiding disease progression (Levin et al., 2022), improving prognosis (Stuver et al., 2022) and neutralizing common sublineages such as Omicron BA.1/2/4/5 (Tuekprakhon et al., 2022).

Ollila et al. (2022) conducted a retrospective study including 37 HM patients who had no serological conversion after a booster. Among the 25 patients injected with Evusheld before SARS-CoV-2 exposure, none were infected, while 25% (3/12) of non-injected patients got infected (*P*=0.007). In a prospective study of 338 HM patients (Zerbit et al., 2022), only 4.9% of the 102 patients who received Evusheld developed COVID-19, significantly lower than the other patients (22%, *P*<0.05). A retrospective multicenter study by Jondreville et al. (2022) evaluated 161 allogeneic hematopoietic stem cell transplantation (allo-HSCT) patients during the Omicron period. The majority of these patients (72.7%) were vaccinated but they exhibited low levels of anti-SARS CoV-2-spike IgG titers (<260 binding antibody units (BAU)/mL). After receiving Evusheld (300mg) as pre-exposure prophylaxis, the SARS-CoV-2 infection rate reduced to 13.7% and no severe cases or death were reported. These studies further highlight that Evusheld can still effectively reduce the risk of COVID-19 infection, severity and mortality in HM patients during the Omicron period, particularly for those with poor vaccine response or unable to receive vaccination (e.g. patients who recently treated with anti-CD20 monoclonal antibody, CAR-T, HSCT, etc.).

In summary, vaccination against SARS-CoV-2 is crucial for protecting HM patients. Considering the low seroconversion rate and declining vaccine effectiveness, it is recommended that HM patients should also receive booster shots and adhere to non-pharmaceutical protective measures such as wearing masks and maintaining social distance, and patients’ families should also receive the initial and booster shots. For patients with low neutralizing antibody titers after booster doses, those who remain seronegative, or who are unable to vaccinate, NmAbs (Evusheld) can provide important protection both before and after exposure. At the same time, to combat the highly mutating SARS-CoV-2 (Vitiello et al., 2022), it is imperative to develop new vaccines and NmAbs that can effectively target multiple SARS-CoV-2 variants (Stuver et al., 2022). This is essential to ensure sustained protection of vulnerable populations and minimize the risk of ongoing transmission and outbreaks.

## 3 Treatment of COVID-19

In addition to the typical symptoms commonly associated with COVID-19, such as fever, cough, dyspnea and fatigue (Piñana et al., 2020; Bird et al., 2021; Yigenoglu et al., 2021), HM patients diagnosed with COVID-19 also exhibit persistent immune dysfunction, delayed or absent seroconversion and delayed viral shedding (Abdul-Jawad et al., 2021), leading to prolonged disease course and a high severity rate. Therefore, treatments that can take into account these unique features of the disease in HM patients may improve their prognosis (García-Suárez et al., 2020).

### 3.1 Antiviral therapy

#### 3.1.1 Small molecular antiviral drugs

Currently, there are three small molecule antiviral drugs available for the treatment of COVID-19, namely, remdesivir, Paxlovid (a combination of nirmatrelvir and ritonavir) and molnupiravir. Remdesivir, an intravenously administered nucleoside analogue, inhibits SARS-CoV-2 RNA polymerase and is more suitable for inpatients (Gottlieb et al., 2022). Molnupiravir is a nucleoside analogue (Jayk Bernal et al., 2022), and nirmatrelvir in Paxlovid is a SARS-CoV-2 main protein (Mpro) inhibitor that increases its blood concentration when combined with the CYP3A4 inhibitor ritonavir (Hammond et al., 2022; Saravolatz et al., 2023), both of which are oral antivirals that are more appropriate for outpatients. Since these small molecule antiviral drugs target sites other than the spike protein, they have been shown to be effective against a variety of SARS-CoV-2 variants *in vitro* (including Omicron BQ.1.1 and Omicron XBB, etc.) (Vangeel et al., 2022; Imai et al., 2023), rendering them important treatment for COVID-19 patients during the Omicron period.

In the general population, Gottlieb et al. (2022), Hammond et al. (2022), and Jayk et al (Jayk Bernal et al., 2022). conducted several randomized, double-blind, controlled trials among unvaccinated outpatients with mild to moderate COVID-19 who were at risk for progression and they have found that early treatment with these drugs (within 5 to 7 days after the onset of symptoms) could significantly reduce the hospitalization and death risks. The efficacy of remdesivir and Paxlovid was found to be superior to that of molnupiravir (87% vs 88% vs 30%). Another randomized controlled trial (WHO Solidarity Trial Consortium, 2022) involving patients hospitalized due to COVID-19 demonstrated that remdesivir reduced the risk for progression (RR=0.88, 95% CI: 0.77~1.00, *P*=0.04) and death (RR=0.86, 95% CI: 0.76~0.98, *P*=0.02) in non-mechanically ventilated patients, but no significant efficacy was observed in those already ventilated (RR=1.13, 95% CI: 0.89~1.42, *P*=0.32). This may be attributed to the fact that severe COVID-19 is mainly characterized by immune response disorder, underscoring the importance of early identification of high-risk patients and prompt administration of antiviral drugs.

Among these antiviral agents, remdesivir has been more frequently used for HM patients with COVID-19, probably owing to its more comprehensive clinical investigations and lower potential for drug interactions. Levy et al. (2021) reported a decreased risk of death in both outpatients and inpatients treated with remdesivir (55/313, 17.6%; OR=0.297, 95% CI: 0.105~0.834, *P*=0.021). Other studies have similarly found that early use of remdesivir (Jaroszewicz et al., 2022; Magyari et al., 2022; Aiello et al., 2023) and molnupiravir (Bołkun et al., 2023) in HM patients with COVID-19 helped to shorten disease course and reduce mortality. Additionally, early antiviral treatment can shorten the duration of viral shedding, lower the risk of COVID-19 progression, and thereby prevent outbreaks among HM patients.

Ritonavir in Paxlovid is a potent inhibitor of CYP3A4 and can interfere with the metabolism of drugs commonly used by HM patients, such as cyclosporine and venetoclax, leading to an increased risk of adverse drug reactions and even life-threating complications (Fishbane et al., 2022). Furthermore, *in vitro* studies have observed that SARS-CoV-2 is capable of developing resistance to remdesivir (Stevens et al., 2022) and nirmatrelvir in Paxlovid through multiple pathways (Iketani et al., 2022b). Molnupiravir can increase the mutation frequency of SARS-CoV-2 RNA (Kabinger et al., 2021) and its safety in HM patients with prolonged viral shedding has yet to be validated. Hence, larger clinical studies with longer follow-up periods are necessary to explore the safety of the above drugs in HM patients with COVID-19.

In summary, small molecule antiviral drugs retain their efficacy against Omicron and early use can significantly decrease the odds of progression and mortality in COVID-19 patients. Additionally, it is imperative for hematologists to thoroughly evaluate contraindications and drug interactions before prescribing these medications to patients with HM and COVID-19 to prevent potential adverse events.

#### 3.1.2 SARS-CoV-2 neutralizing monoclonal antibodies

While the NmAb Evusheld, as mentioned previously, has a prolonged half-life of up to 6 months, making it suitable for both prophylaxis and treatment of COVID-19, most other NmAbs have a shorter half-life and are only appropriate for early treatment after infection. Early use of the NmAbs, such as REGEN-COV (casirivimab-imdevimab) or bamlanivimab, in HM patients with COVID-19 can alleviate symptoms, shorten treatment duration and improve prognosis, but is less effective against variants like Omicron (Weinbergerová et al., 2022). Blennow et al. (2022) have demonstrated that the administration of Evusheld or sotrovimab in HM patients with critical COVID-19 during Omicron can reduce the risk of death (HR=0.13, 95% CI: 0.02~0.61, *P*=0.010). Pagano et al. (2022) investigated the antiviral treatment on 906 patients with hematologic malignancies (HM) who experienced breakthrough COVID-19, with 68.7% of cases attributed to the Omicron infection. The results revealed that the administration of NmAbs alone (50.2% sotrovimab, 38.9% REGEN-COV) or in combination with antiviral agents (50.0% sotrovimab+remdesivir, 28.7% REGEN-COV+remdesivir) was independently associated with a lower mortality (HR=0.155, 95% CI: 0.077~0.313, *P*<0.001; HR=0.407, 95% CI: 0.206~0.803, *P*=0.010).However, further researches have demonstrated that the NmAbs mentioned above exhibit low neutralizing activity against various Omicron subvariants (Iketani et al., 2022a; Takashita et al., 2022). With the increasing prevalence of Omicron-related COVID-19 cases, the risk of hospitalization and death in HM patients treated with NmAbs remains high (Rasmussen et al., 2022). At present, *in vitro* studies have shown that only LY-CoV1404 (bebtelovimab) can neutralize all the sublines (Iketani et al., 2022a) except for Omicron BQ.1.1 and XBB (Imai et al., 2023), but there is still a lack of related clinical studies on HM patients. In general, the neutralizing capacity of most NmAbs against Omicron is both constrained and heterogeneous across its subvariants, indicating that appropriate NmAbs should be selected based on the prevailing variant in the local area.

#### 3.1.3 COVID-19 convalescent plasma

The COVID-19 convalescent plasma (CCP) from recovered patients contains high levels of polyclonal antibodies that have the ability to neutralize SARS-CoV-2 (Ripoll et al., 2022). Early infusion of CCP has been shown to reduce viral load, avoid secondary bacterial and fungal infections and neutralize proinflammatory cytokines in COVID-19 patients (Thompson et al., 2021; Tobian et al., 2022). Additionally, CCP has a low incidence of adverse reactions (Thompson et al., 2021) and is particularly applicable for HM patients with severe humoral immunodeficiency. In a retrospective cohort study (Thompson et al., 2021) involving 966 HM patients with COVID-19 (79.2% had lymphocytic malignancies), the 30-day mortality was significantly lower among the 143 patients who received CCP therapy compared to the untreated control group (13.3% vs 24.8%). A longitudinal cohort and propensity score analysis carried out by Hueso et al. (2022) in patients with B-cell lymphoid disease and COVID-19 showed that CCP significantly reduced mortality in patients who had received anti-CD20 monoclonal antibody treatment. However, another multicenter retrospective study (Levy et al., 2021) involving 313 HM patients with COVID-19 (80.2% had lymphocytic malignancies) did not observe the clinical efficacy of CCP (P>0.100). In conclusion, there is no consensus on whether CCP infusion can benefit HM patients with COVID-19 (Levy et al., 2021; Hueso et al., 2022; Magyari et al., 2022; Ortigoza et al., 2022) due to the great difficulty in CCP preparation, the large differences in neutralizing antibody titers (Joyner et al., 2022) and the different infusion timing and dosage of CCP in different studies. It is noteworthy that the aforementioned studies were conducted prior to the outbreak of Omicron and there is currently no reported clinical study of CCP in HM patients during the Omicron period. However, *in vitro* studies have found that in the Omicron era, the neutralizing antibody titers of CCP obtained from vaccinated COVID-19 patients (Vax CCP) were over 10 times higher than those of regular CCP, with a neutralizing rate of Omicron variant and its subtypes almost close to 100% (Sullivan et al., 2022), and this neutralizing capacity was independent of whether the donor had been previously exposed to the Omicron (Ripoll et al., 2022). These findings suggest that Vax CCP during the Omicron period has potential applications for immunosuppressed patients.

### 3.2 Immunomodulation

#### 3.2.1 Glucocorticoid

Glucocorticoids are known for their robust anti-inflammatory properties, which can help reduce the incidence of respiratory failure and mortality in patients with severe COVID-19. A large randomized controlled trial conducted by Horby et al. (2021) investgated the use of dexamethasone in hospitalized COVID-19 patients and a total of 2104 patients were enrolled and received dexamethasone (6mg, orally or intravenously, once daily, 10 days). The study has found that compared with usual care, dexamethasone use brought signigicant improvements in terms of 28-day mortality in patients requiring invasive mechanical ventilatory support (29.3% vs 41.4%, RR=0.64, 95% CI: 0.51-0.81) and non-invasive mechanical ventilatory support (23.3% vs 26.2%, RR=0.82, 95% CI: 0.72-0.94). But there was no significant relationship between dexamethasone use and mortality in patients without respiratory support. Piñana et al (Piñana et al., 2020). carried out a multicenter retrospective study on 367 HM patients with COVID-19, which showed that low-dose glucocorticoids (intravenous methylprednisolone ≤ 0.5mg/kg/d or equivalent dose of other glucocorticoids) could reduce mortality in HM patients (OR=0.31, 95% CI: 0.11~0.87, *P*=0.020), while doses over 0.5mg/kg/d did not benefit the patients (OR=0.75, 95% CI: 0.34 ~ 1.6, *P*=0.4). Considering the immunocompromised status of HM patients, careful consideration is necessary when determining the timing and dosage of glucocorticoids. It is recommended that when patients present signs of infection aggravation such as decreased oxygen saturation, increased respiratory rate and elevated infection markers, low-dose dexamethasone (Cesaro et al., 2022) should be added to the existing treatment regimen for no longer than 10 days (Horby et al., 2021).

#### 3.2.2 Cytokine receptor antagonist

SARS-CoV-2 infection can trigger a cytokine storm characterized by the production of interleukin (IL)-1, IL-6, tumor necrosis factor (TNF)-α and other cytokines (Huet et al., 2020). A meta-analysis by Peng et al. (2022) indicated that IL-6 receptor antagonists (Tocilizumab, Sarilumab) and IL-1 receptor antagonists (anakinra) could significantly reduce mortality in COVID-19 patients (OR=0.71, 95% CI: 0.57-0.89, *P*=0.004), but the use of tocilizumab poses a potential risk of secondary fungal infection. A randomized controlled study by Kyriazopoulou et al. (2021) and the RECOVERY trial (RECOVERY Collaborative Group, 2021) both showed that anakinra and tocilizumab can significantly ameliorate disease progression and reduce mortality among severe COVID-19 patients. Notably, the majority of patients in these two studies received glucocorticoid treatment (85.9% and 82%, respectively), indicating that the clinical benefits of cytokine receptor antagonists were generated on the basis of glucocorticoid. Therefore, it is recommended to consider cytokine receptor antagonists in patients with persistent hypoxia or inflammatory responses despite glucocorticoid treatment.

However, researches on cytokine receptor antagonists in HM patients remain relatively limited, with a primary focus on tocilizumab. The lack of standardized reference guidelines for the indications, timing, dosage and combination therapy of tocilizumab across diverse studies has contributed to varying conclusions. In several case reports (Zhang et al., 2020; Bouchlarhem et al., 2022), the addition of IL-6 pathway inhibitor (tocilizumab) to glucocorticoids improved hypoxia and prognosis in HM patients (including those with CML or MM) with severe COVID-19, and short-term use would not increase the risk of secondary infection (Frigault et al., 2020). But in a retrospective study (García-Suárez et al., 2020) involving 692 patients with HM and COVID-19, 318 (46%) patients received systemic corticosteroids and 132 (19%) patients received tocilizumab. This study demonstrated an association between tocilizumab administration and an increased mortality rate in HM patients with mild to moderate COVID-19 (HR=5.94, 95% CI: 1.80~19.6, *P*=0.002), while no significant difference was observed in severe/critical patients (HR=0.87, 95% CI: 0.62~1.23, *P*=0.40). These findings suggest that tocilizumab may have potential utility in mitigating hyperactive cytokine responses specifically in severe/critical COVID-19 cases. Nevertheless, given the complex cytokine and inflammatory pathways and the intricate immunological conditions in HM patients, randomized controlled trials are warranted to elucidate the specific roles of tocilizumab and other cytokine receptor antagonists in patients with HM and COVID-19.

#### 3.2.3 Kinase inhibitor

Janus tyrosine kinase (JAK) inhibitors can alleviate the hyperinflammatory state secondary to COVID-19 by blocking multiple cytokine signaling pathways. A meta-analysis of eight randomized controlled trials on the effectiveness of using JAK inhibitors such as baricitinib and ruxolitinib in patients with severe COVID-19. It was found that JAK inhibitors can reduce the risk of death by 43% (RR 0.57, 95% CI 0.45-0.72, *P*<0.0001) *(* RECOVERY Collaborative Group, 2022b). Baricitinib can also exert antiviral effects through other mechanisms (Jorgensen et al., 2020) and is the only JAK inhibitor that has been proven to be effective in reducing mortality in COVID-19 patients in randomized clinical trials, usually used in combination with glucocorticoids. The Randomized Evaluation of COVID-19 Therapy (RECOVERY) trial (RECOVERY Collaborative Group, 2022b), conducted in 2021 before the Omicron period, was an investigator-initiated, individually randomized, controlled, open-label, platform trial that enrolled 8,156 hospitalized COVID-19 patients. Patients were randomly assigned to receive either baricitinib plus usual care (50.86%) or usual care alone (49.14%), with 95% of patients receiving glucocorticoids and 23% receiving tocilizumab. The 28-day mortality of patients in the combination treatment group was lower than that in the usual care group (12% vs 14%, aRR=0.87, 95% CI: 0.77-0.99, *P*=0.028). A randomized, double-blind, placebo-controlled trial by Kalil et al. (2021) demonstrated that the combination of baricitinib and remdesivir can shorten the course of COVID-19 and reduce adverse reactions, especially for severely ill patients requiring oxygen support. Similar findings were also reported by other studies, which confirmed that baricitinib can reduce the 28-day mortality in hospitalized COVID-19 patients (Marconi et al., 2021), and patients with critical infection can also benefit from it (Ely et al., 2022), but it is still unclear whether the use of baricitinib in HM patients with COVID-19 is both safe and effective.

In addition to baricitinib, a randomized, double-blind, placebo-controlled clinical trial carried out by Aman et al. (2021) preliminarily confirmed that imatinib, an oral TKI commonly used in CML patients, could reduce mortality among severe COVID-19 patients. The relatively lower SARS-CoV-2 infection rate and mortality of CML patients may be linked to the potential protective effects of TKIs (García-Suárez et al., 2020; Morales-Ortega et al., 2021). Ruxolitinib, as one of the most well-established JAK inhibitors, has garnered widespread attention in HM patients afflicted with SARS-CoV-2. Barbui et al. (2021) observed that discontinuing ruxolitinib in MPN patients is associated with an increased risk of mortality. Several case reports indicated that MPN (Koschmieder et al., 2020) and CML (Innes et al., 2020) patients using ruxolitinib have experienced a favorable clinical course of COVID-19. Furthermore, other small cases series (Vannucchi et al., 2021) and a meta-analysis (Patoulias et al., 2021) also suggested its potential to improve the prognosis of COVID-19 patients. However, two phase 3 randomized, placebo-controlled clinical trials with the use of ruxolitinib failed to reach their primary endpoints. Hence, further clinical investigations are imperative to comprehensively assess the role of kinase inhibitors in HM patients with COVID-19.

In summary, since HM patients are rarely included in the efficacy assessment of anti-COVID-19 interventions, there are insufficient relevant data to draw definitive conclusions. Nevertheless, available evidence suggests that small molecule antiviral medications can be used in HM patients with mild to moderate COVID-19 during the early stage of infection. Moreover, HM patients with severe immune impairment such as those recently undergone CAR-T, HSCT or B-cell depletion therapy may benefit from the administration of appropriate NmAbs at the early stage based on the dominant epidemic variants or transfusion of high titer CCP to improve neutralizing antibody levels and accelerate virus clearance. For HM patients with moderate to severe COVID-19, low-dose glucocorticoids are recommended in combination with antiviral therapy. In cases where the aforementioned therapies prove ineffective, the addition of cytokine receptor antagonists or kinase inhibitors may be considered, but it is not advisable to use both concurrently. Close monitoring of patients’ vital signs and infection indicators is crucial during the administration of these medications (Cesaro et al., 2022).

## 4 Treatment strategy of blood disease

### 4.1 Chemotherapy, targeted treatment and other treatment programs

Most studies conducted both before and after the prevalence of Omicron suggested a weak association between hematological treatments (including chemotherapy, targeted therapy and demethylation therapy) and unfavorable outcomes (Pagano et al., 2022). This may be attributed to treatment-induced immune system anergy, which prevents SARS-CoV-2 from inducing a high inflammatory response. Moreover, some immunosuppressive drugs used to treat hematological diseases have proven effective in treating COVID-19 (Vijenthira et al., 2020; Bird et al., 2021). In a similar vein, the implementation of improved post-infection monitoring and treatment measures may also have contributed to these findings.

During the Omicron period, the negative impact of recent anti-CD20 monoclonal antibody use on the prognosis of patients with HM and COVID-19 has diminished compared to previous periods (Blennow et al., 2022; Bronstein et al., 2022; Kohn et al., 2022; Shaw et al., 2022; Bołkun et al., 2023) (**Table 1**), which may be attributed to several factors, including the decreased virulence of Omicron, the general vaccination of HM patients, the prompt application of antiviral medications, and the extended time interval between anti-CD20 administration and SARS-CoV-2 infection, which may have mitigated the adverse effects of pre-infection anti-CD20 monoclonal antibodies administration on patients.

**Table 1:** Omicron variant infection in patients with hematological malignancies.

reference	Total (N)	Age, (IQR)/[range]	Clinical characteristics	Mortality (%)	Omicron related mortality (%)	Vaccination (%)	Inpatient (%)	Clinical Severity	Risk factors for hospitalization/death	Protective/irrelevant factors
(Pagano et al., 2022)	1548	66 (55-75)	68.7% omicron 76% lymphoid malignancies 23.0% myeloid malignancies 0.7% AA	149/1548 (9.2)	41/517 (7.9)	Vaccinated (100)	53.2	18.3% asymptomatic 39.0% mild 32.9% severe 9.8% critical	Older age (HR 1.042) Active disease (HR 1.981) ≥ 2 comorbidities (HR 1.503)	Protective: NmAb (HR 0.155) NmAb + antivirals (HR 0.407) Irrelevant: Treatment of HM
(Kohn et al., 2022)	63	66 (21–97)	54.0% omicron 46.0% unknown 82.5% lymphoma 17.5% CLL	4/63 (6.3)	3/34 (8.8)	Vaccinated (95.2) ≥ 3 doses (76.2) 2 doses (17.5)	33.3	14.3% asymptomatic 52.4% mild 33.3% severe	Refectory disease (P<0.001) Age ≥ 70 (P=0.016)	Irrelevant: Anti-CD20 Mab (P=0.59)
(Bronstein et al., 2022)	131	68 (57-68)	100% omicron 45.0% NHL 27.5% MM 27.5% CLL	7/131 (5.3)	7/131 (5.3)	≥ 3 doses (87.1)	26.7	85.5% mild/moderate 14.5% severe/critical	CLL (OR 2.7) Active hematologic treatment (OR 2.9)	Irrelevant: Anti-CD20 Mab NmAb
(Blennow et al., 2022)	593	66 (54-75)	100% omicron 70.3% lymphoproliferative malignancies 26.0% myeloproliferative malignancies 3.7% other	54/593 (9.1)	54/593 (9.1)	Vaccinated (83.1) ≥ 2 doses (78.6) 1 dose (4.6)	52.1	8.9% critical	Older age (HR 1.044) Active malignancy (HR 2.473)	Protective: Booster shot (HR 0.286) Sotrovimab, Evusheld (HR 0.13) Irrelevant: Anti-CD20 Mab
(Shaw et al., 2022)	75	70 [18-91]	72.0% omicron 44.0% lymphoma 33.3% MM 10.7% myeloproliferative malignancies 8.0% MDS 4.0% AL	4/75 (5.3)	-	Vaccinated (86.7) 2 doses (57.3) 3 doses (25.3)	49.3	-	Age ≥ 70 (RR 5.9)	Protective: Omicron BA.1 infection (RR 0.5) ≥ 2 doses of vaccine (RR 0.5) Irrelevant: Anti-CD20 Mab Sotrovimab
(Rasmussen et al., 2022)	1104	70 (59-77)	66.6% omicron 9.9% delta 23.5% unknown	96/1104 (8.7)	-	Vaccinated (100) 2 doses (10.1) ≥ 3 doses (86.0)	36.1	-	Age ≥ 65 (aHR 7.11) Delta variant infection (aHR 1.71)	Protective: Booster shot (aHR 0.26-0.37)
(Martin-Onraët et al., 2022)	115	50 (35-63)	100% omicron 52.2% NHL 14.0% ALL 10.4% AML 10.4% HL 7.8% myeloma/plasmacytoma 3.5% CLL 1.7% MDS	9/115 (7.8)	9/115 (7.8)	Vaccinated (77.2) 2 doses (43.8) 3 doses (20.2)	46.1	52.6% mild 9.7% moderate 28.0% severe/critical	-	Irrelevant: Remdesivir
(Bołkun et al., 2023)	175	56 (18–86)	100% omicron 45.1% lymphoma 33.7% MDS/AML 21.1% MM	7/175 (4.0)	7/175 (4.0)	Vaccinated (77.1) 3 doses (49.7)	20.0	-	ECOG <2 (P=0.0007) Hb <10 g/dL (P=0.0385)	Irrelevant: Anti-CD20 Mab (P=0.987) Conventional chemotherapy (P=0.872) Type of HM (P=0.282)
(Yan et al., 2022)	156	62 [12-91]	100% omicron 42.3% leukemia/MDS 39.7% lymphoma 17.9% myeloma/amyloidosis	4/156 (2.3)	4/156 (2.3)	Vaccinated (80.8)	18.6	-	Anti-CD20 Mab (OR 5.59) Relapse/refractory disease (OR 5.69)	Protective: Early sotrovimab Irrelevant: Systemic steroids (P=0.631)
(Cattaneo et al., 2023)	94	-	84.2% omicron 40.4% lymphoma 18.1% MDS-MPS 14.9% MM 12.8% AL 13.8% other	11/94 (11.7)	-	Vaccinated (91.5) 3 doses (41.5)	42.6	-	Anti-CD20 Mab	Irrelevant: Vaccination
(Mikulska et al., 2023)	328	66 [16-89]	94.2% omicron 33.8% NHL 23.8% MM 19.5% AL 10.1% CLL 2.7% MDS 10.1% other	18/328 (5.5)	7/309 (2.3)	Vaccinated (91.7) ≥ 3 doses (65)	12.8	-	Older age (HR 1.056) AML/MDS (HR 5.172)	Protective: Omicron infection (HR 0.237)
(Zhu et al., 2023)	412	48 (39-64)	100% omicron 38.3% AL 24.5% CML 21.8% plasma cell disorders 12.6% lymphoma/CLL 1.5% MDS 1.2% other	9/412 (2.2)	9/412 (2.2)	Vaccinated (65.3)	100	94.7% mild/moderate 5.3% severe/critical	Age ≥ 65 (HR 2.941) Comorbidities (HR 5.581) Active disease (HR 2.428)	Irrelevant: Type of HM (P=0.077)

AA, aplastic anemia; NmAb, neutralizing monoclonal antibody; CLL, chronic lymphocytic leukemia; MM, multiple myeloma; MDS, myelodysplastic syndrome; AL, acute leukemia; NHL, non-Hodgkin’s lymphoma; ALL, acute lymphoblastic leukemia; AML, acute myeloid leukemia; HL, Hodgkin’s lymphoma; ECOG, Eastern Cooperative Oncology Group score standard; Hb, hemoglobin; MDS-MPS, myelodysplastic‐myeloproliferative syndromes.

However, a prospective study conducted by Zerbit et al. (2022) of 338 HM patients who were not yet infected with SARS-CoV-2 during the Omicron period found a higher infection rate among patients who had recently received immunotherapy (anti-CD20 monoclonal antibody, Bruton’s tyrosine kinase inhibitor (BTKi), etc.) (85.5% vs 41.0%, P<0.0001). In addition, Leuva et al. (2022) carried out a large prospective cohort study in cancer patients (13.6% HM patients, including Delta and Omicron variant) and found that recent chemotherapy (HR 2.993, 95% CI: 2.484-3.607, P<0.0001) and targeted treatments (including proteasome inhibitors, anti-CD20 monoclonal antibodies, anti-CD38 monoclonal antibodies) (HR 1.781, 95% CI: 1.546-2.051, P<0.0001) resulted in higher infection and death rates in HM patients with diagnoses of CLL, other leukemia, lymphoma, and myeloma. In summary, although previous hematology-related treatments have a low impact on the prognosis of HM patients with COVID-19, treatment regimens such as high-intensity chemotherapy and anti-CD20 monoclonal antibodies can severely suppress patients’ cellular or humoral immunity, increasing the risk of SARS-CoV-2 infection and death. Therefore, when formulating a treatment regimens for HM patients who are not currently infected with SARS-CoV-2, priority should be given to less toxic and less immunosuppressive drugs, taking into account the patient’s general condition, vaccination status, availability of medical resources and local prevalence of COVID-19. Additionally, abrupt interruption of hematological treatment may lead to disease relapse and increase the risk of death in HM patients with COVID-19 (Pinato et al., 2021; Jain et al., 2022). So a gradual reduction of drug doses can be adopted to reduce myelosuppression and improve drug-induced organ toxicity in HM patients who are diagnosed with COVID-19 during hematological treatment when necessary, but immediate discontinuation of treatment is not advisable (Fox et al., 2020; García-Suárez et al., 2020; Jee et al., 2020; Kuderer et al., 2020; Lee et al., 2020; Paul et al., 2021).

Patients with AML, ALL and CLL who are also diagnosed with COVID-19 have presented higher mortality rates, so chemotherapy should be postponed if the patient’s condition permits (Chatzikonstantinou et al., 2021a; Núñez-Torrón et al., 2021; Palanques-Pastor et al., 2021; Marchesi et al., 2022). If delaying is not feasible due to the progression of HM, AML and ALL patients aged < 60 years may consider using standard chemotherapy regimens (Paul et al., 2021). However, if circumstances do not allow (such as intolerance or limited medical resources) or if they are over 60 years of age, low-intensity chemotherapy or reduced chemotherapy doses should be applied (American Society of Hematology, 2022).

Patients with CLL should avoid using anti-CD20 monoclonal antibodies to prevent serious immunosuppression (Chatzikonstantinou et al., 2021a). Furthermore, Scarfò et al. (2020) demonstrated a potential benefit of Bruton’s tyrosine kinase inhibitor (BTKi) in reducing the hospitalization rate of CLL patients with severe COVID-19, but subsequent research by Chatzikonstantinou et al. (2021b) indicated that CLL-related treatments, including BTKi, venetoclax and anti-CD20 monoclonal antibodies, were associated with a higher risk of death (OR = 2.13, 95% CI: 1.44 to 3.20, P < 0.001). Conversely, a recent study by Roeker et al. (2021) found no association between hypogammaglobulinemia (P=0.78) or CLL-related treatment (including BTKi, etc.) (P>0.2) and outcomes in patients with CLL and COVID-19. Overall, apart from avoidance of anti-CD20 monoclonal antibodies, there is currently no clear evidence to support a change of hematological regimen in CLL patients with COVID-19 and treatment decisions should be made on a case-by-case basis (American Society of Hematology, [[NoYear]]a; Sharafeldin et al., 2022).

For lymphoma patients, recent anti-CD20 monotherapy has conferred an excess risk of hospitalization and death (Shafat et al., 2022; Yan et al., 2022; Cattaneo et al., 2023). Although several studies have found that recent anti-CD20 monotherapy has little impact on patient outcomes (Blennow et al., 2022; Bronstein et al., 2022; Kohn et al., 2022; Shaw et al., 2022), the timing of anti-CD20 monotherapy use in these studies was mostly 6-12 months prior to SARS-CoV-2 infection rather than during confirmed COVID-19 period. Therefore, lymphoma patients with COVID-19 should avoid anti-CD20 monotherapy and temporarily suspend anti-lymphoma therapy when the infection worsens (Passamonti et al., 2022). What is more, lymphoma patients who have recently undergone anti-CD20 treatment have low seroconversion rates after vaccination and it is suggested that these patients receive revaccination 6 months after the end of treatment (Haggenburg et al., 2022). However, neutralizing antibodies can persist in patients receiving anti-CD20 antibodies after immunizing (Shree et al., 2022), so clinicians should carefully evaluate whether to administer anti-CD20 antibodies or vaccinate lymphoma patients in advance.

Patients with high-risk MDS diagnosed with COVID-19 are recommended to initiate regular-dose demethylation therapy immediately without delay or dose adjustment, while patients with low-risk MDS are advised to delay or reduce the frequency of therapy (American Society of Hematology, [[NoYear]]b). As previously described, treatment with TKIs was associated with milder COVID-19 in patients with CML or MPN and abrupt discontinuation of the TKI (ruxolitinib) could increase the risk of death in MPN patients increased instead (OR=8.51, 95% CI: 1.14-63.4, P=0.037) (Barbui et al., 2021). Hence, patients with CML or MPN diagnosed with COVID-19 do not need to delay or interrupt treatment after the onset of COVID-19, but should discontinue TKIs when infection symptoms and respiratory distress worsen.

**Figure 1:**
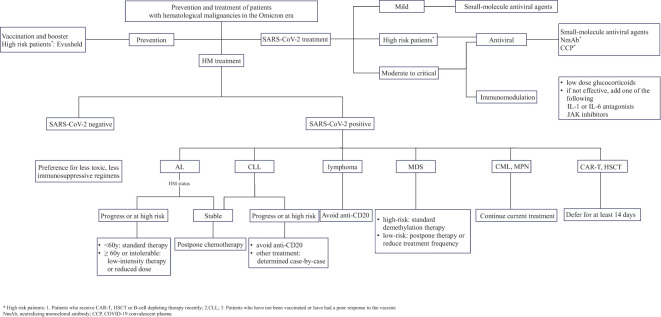
Prevention and treatment of patients with hematological malignancies in the Omicron era.

In brief, patients with distinct types of HM ought to adopt different strategies of discontinuing or modifying treatment amidst the Omicron wave (**Figure 1**). Prior to initiating hematological disease treatment, a personalized approach to treatment and medication scheduling should be taken based on a comprehensive assessment of the patient’s vaccination status, susceptibility to SARS-CoV-2 infection, prior medication history and disease severity, which will help minimize treatment interruptions and optimize outcomes.

### 4.2 HSCT and CAR-T therapy

Patients with HM usually require high-dose chemotherapy and long-term immunosuppressive therapy before and after HSCT or CAR-T therapy, which amplifies the risk of SARS-CoV-2 infection and death. Before the prevalence of Omicron variant, the mortality of COVID-19 patients who underwent allo-HSCT was slightly higher than that of patients who received autologous HSCT (auto-HSCT) (18.5%-35.5% vs 14.3%-33.3%) (Passamonti et al., 2020; Piñana et al., 2020; Varma et al., 2020; Mushtaq et al., 2021; Pagano et al., 2021; Sharma et al., 2021; Busca et al., 2022). Additionally, post-transplant immunosuppressive therapy further increased the death risk (Altuntas et al., 2021). Nevertheless, Pagano et al. (2021) and Piñana et al. (2020) have discovered that the mortality of patients receiving auto-HSCT or allo-HSCT was slightly lower than that of non-HSCT patients (*P*<0.03), which could be explained by factors such as younger age, longer post-transplant periods, fewer comorbidities and better control of blood diseases in the transplantation group (Karhana et al., 2023). In contrast, CAR-T therapy has conferred a statistically significant excess risk of death in COVID-19 patients compared to HSCT (Busca et al., 2022), which can be ascribed to long-term B-cell exhaustion, hypogammaglobulinemia, loss of T-cell repertoire diversity (Jarisch et al., 2022) and exacerbation of cytokine storm caused by COVID-19 (Luque-Paz et al., 2022). During the Omicron wave, the mortality of HSCT and CAR-T patients with COVID-19 significantly decreased compared to the pre-Omicron period (Pagano et al., 2022). With the implantation of appropriate prophylactic and therapeutic measures, such as Evusheld injection (Jondreville et al., 2022) and re-vaccination after transplantation (Haggenburg et al., 2022), the mortality of HSCT patients with COVID-19 has been reduced to 0%, although that of CAR-T patients remains high, ranging from 20-25% (Jarisch et al., 2022; Pagano et al., 2022).

In summary, the mortality of HM patients undergoing CAR-T or HSCT and infected with Omicron was significantly lower than before, but CAR-T patients still experienced a higher mortality rate than others. Therefore, it is crucial to strengthen preventive and treatment measures for these vulnerable patients. Revaccination should be carried out 3-6 months after cell infusion (Perram et al., 2022), irrespective of their vaccination status before treatment. Passive immunotherapy such as NmAbs and CCP should be actively utilized according to the patient’s specific condition (Luque-Paz et al., 2022). HM patients who have already contracted SARS-CoV-2 should carefully consider CAR-T therapy or select the appropriate timing for HSCT, and it is recommended to delay HSCT for at least 14 days until symptoms of infection have significantly improved (Dioverti et al., 2022).

## 5 Conclusion

HM patients should weigh the severity of their hematological disease and the risk of COVID-19 infection before initiating HM-related treatment and receive full vaccination and booster shots prior to treatment if possible. Patients with suboptimal vaccine response or who are unable to vaccinate may consider injecting NmAbs (Evusheld). For HM patients with COVID-19, those with mild to moderate infection are recommended to use small molecule antiviral drugs early, while those with severe immunodeficiency may benefit from the addition of NmAbs and CCPs early. Those with moderate to severe infection can be treated with low-dose glucocorticoids and if the steroid therapy is ineffective, a combination with JAK inhibitors (baricitinib) or cytokine receptor antagonists can be used under close monitoring to enhance immunoregulation. In terms of the management of HM itself, given the heterogeneity of HM patients with different types, individualized treatment plans should be tailored based on their general condition, medical history, HM progression and infection severity. And it is imperative to initiate prompt and effective treatment for HM progression and not to defer it due to COVID-19.

Although the current vaccine based on SARS-CoV-2 wild-type is still effective against the Omicron variant, as well as herd immunity can be established after natural Omicron infection, the potential immune evasion of SARS-CoV-2 due to factors such as therapeutic drugs screening and prolonged virus shedding in HM patients may contribute to the emergence of novel mutant strains. Therefore, small molecule antiviral drugs that target alternative viral components beyond the S protein and Vax-CCP containing polyclonal antibodies are expected to be important antiviral strategies. Furthermore, it is also especially important to reinforce non-pharmaceutical protection measures, establish vaccines and antiviral medications for new targets, and monitor mutant strains in immunocompromised populations.

## Author contributions

WG wrote the review. YZ searched comprehensive literature. SFrevised the review.. All authors contributed to the article and approved the submitted version.
